# LungPanelNet: a machine learning-based approach for the early prediction and differentiation of non-small cell lung cancer

**DOI:** 10.3389/fonc.2025.1702589

**Published:** 2026-01-13

**Authors:** Li Zhao, Mei Li, Juju Qi, Lingling Wan

**Affiliations:** Department of Clinical Laboratory, Shijiazhuang People’s Hospital, Shijiazhuang, Hebei, China

**Keywords:** classification, early diagnosis, LungPanelNet, machine learning, non-small cell lung cancer, serum biomarkers, tumor markers

## Abstract

Non-small cell lung cancer (NSCLC) represents a major global health challenge, primarily due to its frequent diagnosis at advanced stages, which significantly limits therapeutic efficacy and results in poor survival outcomes. A critical unmet need exists for non-invasive, accurate diagnostic tools for early detection. Objective: This study aimed to develop and validate a robust machine learning model based on a panel of serum tumor markers for the early prediction of NSCLC and its differentiation from benign pulmonary conditions. Methods: In this retrospective cohort study, we recruited 2,283 participants, including 1,339 with NSCLC, 313 with pneumonia, 260 with biopsy-confirmed benign lesions, and 371 with other benign lung masses. Serum levels of six key tumor markers—Squamous Cell Carcinoma Antigen (SCCA), Carcinoembryonic Antigen (CEA), Cancer Antigen 125 (CA-125), Cytokeratin 19 Fragment (CYFRA21-1), Neuron-Specific Enolase (NSE), and Pro-Gastrin-Releasing Peptide (ProGRP)—were quantified, and a custom deep neural network, LungPanelNet, was constructed for the classification task. Results: The model demonstrated superior predictive performance on an independent testing set, achieving an area under the receiver operating characteristic curve (AUC-ROC) of 0.92 (95% CI: 0.88–0.96), with an accuracy of 89.3%, a sensitivity of 91.5%, and a specificity of 87.8%. Feature importance analysis identified SCCA and CYFRA21-1 as the most significant predictors. Conclusion: Our findings demonstrate that a machine learning model integrating a panel of serum tumor markers can effectively distinguish NSCLC from a spectrum of benign pulmonary conditions with high accuracy. This approach shows promise as a clinical decision-support tool, though further validation in larger, prospective, multi-center cohorts is warranted. This was a retrospective cohort study without clinical trial registration.

## 1 Introduction

Lung cancer remains the leading cause of cancer-related mortality worldwide, posing a significant public health burden. According to global cancer statistics, it is responsible for approximately 1.8 million deaths annually, more than any other malignancy (1, 2). Non-small cell lung cancer (NSCLC) is the predominant histological subtype, accounting for approximately 85% of all lung cancer cases (3). The primary challenge in managing NSCLC lies in its insidious onset and the lack of specific symptoms in its early stages. Consequently, a substantial proportion of patients, estimated between 49-53%, are diagnosed at an advanced or metastatic stage (Stage IV), where treatment options are largely palliative and the five-year survival rate is dismally low, often below 5% (4, 5). In stark contrast, patients diagnosed with localized, early-stage disease can achieve five-year survival rates of up to 70-90% following surgical resection, underscoring the critical importance of early detection in improving patient prognosis and long-term outcomes (6).

The current diagnostic gold standard for NSCLC relies on histopathological examination of tissue specimens obtained through invasive procedures such as bronchoscopy or transthoracic needle biopsy. While these methods offer definitive diagnoses, they are not without significant limitations. They are invasive, carry risks of complications like pneumothorax and bleeding, and may not be feasible for all patients, particularly those with comorbidities or centrally located tumors (7). Furthermore, imaging techniques such as low-dose computed tomography (LDCT) screening, while crucial for initial detection, often suffer from a lack of specificity. Many benign pulmonary conditions, including pneumonia, granulomas, and inflammatory masses, can mimic the radiological appearance of malignant nodules, leading to a high false-positive rate (8). This can result in unnecessary anxiety for patients and lead to further invasive, costly, and potentially harmful diagnostic workups, an issue highlighted by the National Lung Screening Trial which reported a high rate of false positives (9, 10). Therefore, there is a pressing and urgent need for novel, non-invasive, and more reliable biomarkers and diagnostic tools to facilitate the early and accurate management of patients with pulmonary nodules (11, 12).

In this context, liquid biopsy, particularly the analysis of serum tumor markers, has emerged as a highly promising avenue for cancer diagnostics. These biomarkers are substances, often proteins, released by tumor cells or by the body in response to the presence of cancer, which can be measured in peripheral blood (13). This approach offers a minimally invasive, cost-effective, and easily repeatable method for screening and diagnosis. A panel of tumor markers has shown potential in the context of lung cancer. Squamous cell carcinoma antigen (SCCA) is primarily associated with squamous cell carcinoma, a major subtype of NSCLC (14). Carcinoembryonic antigen (CEA) is a well-established, albeit non-specific, marker used in the monitoring and prognostic assessment of various cancers, including lung cancer (15). Cancer antigen 125 (CA-125), though traditionally linked to ovarian cancer, has been found to be elevated in a subset of NSCLC patients. Cytokeratin fragment 21-1 (CYFRA21-1), a fragment of cytokeratin 19, has demonstrated high specificity for NSCLC and is considered one of the most reliable markers for this disease (16). Neuron-specific enolase (NSE) and pro-gastrin-releasing peptide (ProGRP) also serve as valuable markers in the differential diagnosis of pulmonary masses.

Furthermore, while Neuron-specific enolase (NSE) and pro-gastrin-releasing peptide (ProGRP) are recognized as valuable markers for SCLC, their inclusion in our panel is strategic. It enables the model to leverage their high specificity for SCLC to aid in the critical clinical task of differentiating NSCLC from other malignancies, thereby refining the overall diagnostic accuracy within a multi-class framework.

While individual tumor markers often lack the required sensitivity and specificity for standalone diagnostic use, the simultaneous evaluation of a multi-marker panel may provide a more comprehensive and accurate reflection of the underlying pathological processes. However, interpreting the complex, non-linear relationships within a high-dimensional biomarker dataset presents a significant challenge for traditional statistical methods. This is where machine learning (ML) techniques offer a transformative solution. ML algorithms are exceptionally adept at identifying subtle patterns and complex interactions in large datasets that may be imperceptible to human analysis or conventional statistics (17). By leveraging sophisticated architectures, it is possible to develop predictive models that integrate multiple data points to generate a single, probabilistic risk score. Such data-driven approaches are already demonstrating success in classifying NSCLC subtypes and predicting outcomes in other cancers by analyzing dynamic biomarker changes over time (8, 18–20). In this study, we hypothesize that a custom deep neural network, LungPanelNet, integrating a panel of six serum tumor markers, can accurately predict the presence of NSCLC and differentiate it from common benign pulmonary conditions. The objective of this research is twofold: first, to analyze the serum profiles of these six markers in a well-characterized cohort; and second, to develop and validate a robust predictive tool for the early detection of NSCLC. Through this research, we aim to contribute to the development of a non-invasive screening strategy that could ultimately enhance early diagnosis, guide clinical decision-making, and improve patient outcomes in the fight against lung cancer.It is important to clarify that the intended clinical application of the LungPanelNet model is not for primary screening of an asymptomatic, healthy population, but rather to aid in the differential diagnosis for patients who have already been found to have radiological lung abnormalities, such as indeterminate pulmonary nodules.

## 2 Materials and methods

### 2.1 Study design and participants

**Figure 1:**
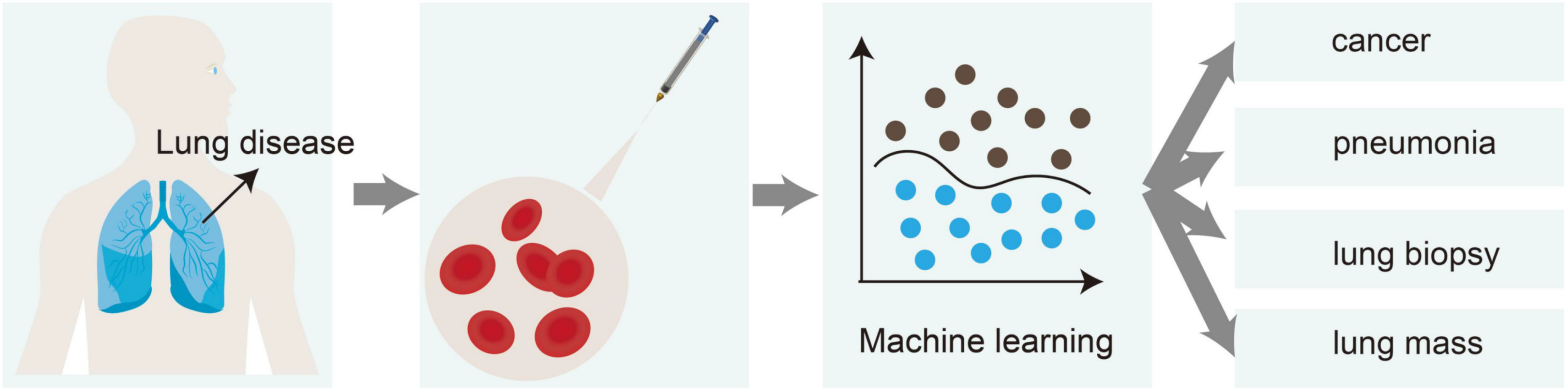
Workflow chart of the study.

The overall study workflow is depicted in **Figure 1**. This retrospective cohort study enrolled 2,284 participants, stratified into four diagnostic groups. The study focused on differentiating Non-Small Cell Lung Cancer (NSCLC) from benign conditions; thus, other cancer types (e.g., SCLC) were excluded. Demographic characteristics are summarized in **Table 1**.

**Table 1:** Dataset demographic characteristics.

Characteristic Category	Cancer	Pneumonia	Biopsy-confirmed benign lesions	Lung mass
Age (years)	25-91	14-93	33-90	22-87
Gender				
Male	819 (61.12%)	54 (49.20%)	156 (60.00%)	173 (46.63%)
Female	521 (38.88%)	159 (50.80%)	104 (40.00%)	198 (53.37%)
Smoking history				
Never-Smokers	648(48.36%)	135(63.38%)	93(35.77%)	176(46.63%)
Former Smokers	56(4.18%)	23(10.80%)	14(5.38%)	32(8.63%)
Current Smokers	636(47.46%)	55(25.82%)	153(58.85%)	163(43.94%)

### 2.2 Serum biomarker analysis

**Figure 2:**
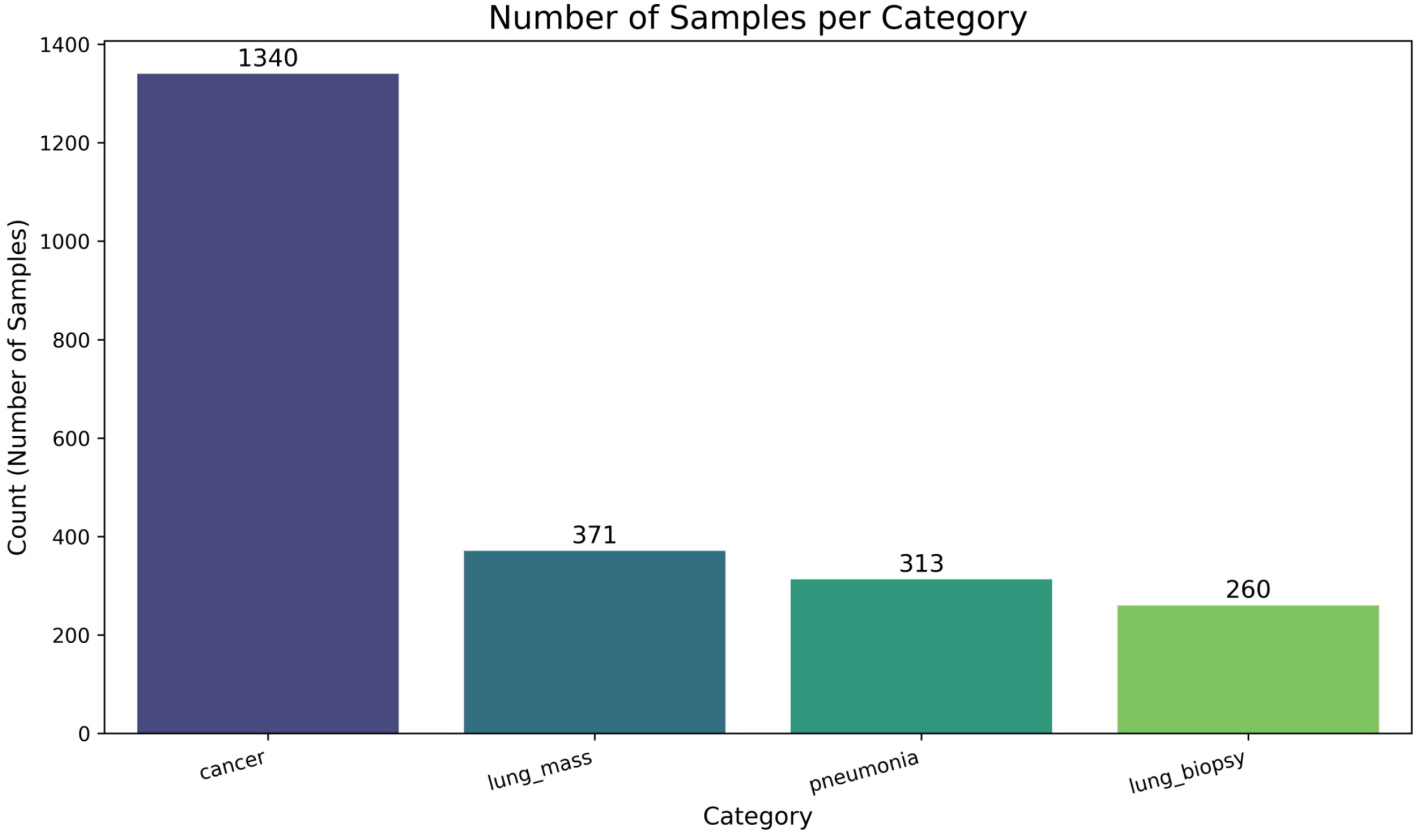
Dataset classes count.

Peripheral blood was collected from all participants. Serum levels of six biomarkers—Carcinoembryonic Antigen (CEA), Squamous Cell Carcinoma Antigen (SCCA), Cytokeratin Fragment 21-1 (CYFRA21-1), Neuron-Specific Enolase (NSE), Pro-Gastrin-Releasing Peptide (ProGRP), and Cancer Antigen 125 (CA-125)—were quantified using enzyme-linked immunosorbent assay (ELISA). Their expression patterns across groups are visualized in a clustered heatmap (**Figure 2**).

### 2.3 Machine learning model development and evaluation

The dataset, comprising the six biomarker values for all participants, was randomly split into a training set (70%) and a hold-out test set (30%), with stratification by diagnostic category.

A custom deep neural network, LungPanelNet, was developed to perform the four-class classification. The model was designed to capture complex, non-linear interactions between the serum biomarkers. Key architectural components included multiple fully-connected layers with ReLU activation, Batch Normalization, and Dropout for regularization. (Detailed architecture and hyperparameters are provided in the Supplementary Methods.

Model performance was evaluated on the independent test set using accuracy, sensitivity, specificity, F1-score, and area under the receiver operating characteristic curve (AUC-ROC). For benchmarking, LungPanelNet was compared against conventional classifiers, including Naïve Bayes, k-Nearest Neighbors, Support Vector Machine, and Logistic Regression.

### 2.4 Ethical considerations

The study protocol was reviewed and approved by the institutional review board (IRB). Due to the retrospective nature of the study and the use of de-identified data, the requirement for individual informed consent was waived. All procedures adhered strictly to the ethical standards outlined in the Declaration of Helsinki and its subsequent amendments.

### 2.5 Machine learning model development

#### 2.5.1 Data preprocessing and splitting

The core of our diagnostic pipeline is a machine learning model, as depicted in **Figure 1**. The dataset, containing the six quantified biomarker values for all 2,284 participants, was prepared for model training. The data was randomly partitioned into a training set (70%) and an independent testing set (30%).

#### 2.5.2 Model architecture: LungPanelNet

For the classification task, we developed a custom deep neural network, which we have named LungPanelNet. The architecture of LungPanelNet is a feed-forward network defined as follows:

- An input layer that accepts the six serum biomarker concentrations.
- A first hidden layer consisting of 128 neurons, followed by a Batch Normalization layer (BatchNorm1d), a Rectified Linear Unit (ReLU) activation function, and a Dropout layer with a 50% rate to prevent overfitting.
- A second hidden layer with 64 neurons, similarly followed by Batch Normalization, a ReLU activation, and a 50% Dropout layer.
- An output layer with a linear activation that produces predictions for the four diagnostic classes.

The Rectified Linear Unit (ReLU) activation function was chosen for its effectiveness in mitigating the vanishing gradient problem and enabling efficient training. While ReLU itself is a piecewise linear function, its application following multiple fully-connected layers allows the network to approximate complex, non-linear decision boundaries, which is essential for capturing the intricate interactions between the serum biomarkers.

This multi-layered architecture, coupled with non-linear activations, is specifically designed to capture these intricate relationships. The inclusion of Batch Normalization and aggressive Dropout are critical regularization strategies to mitigate the risk of overfitting, ensuring the model generalizes well to unseen data despite its capacity for learning complex functions.

#### 2.5.3 Model training and evaluation

The LungPanelNet model was trained exclusively on the training set using a standard backpropagation algorithm. Model performance was rigorously evaluated on the unseen testing set. Key evaluation metrics included Accuracy, Sensitivity, Specificity, F1-Score, and the Area Under the Receiver Operating Characteristic Curve (AUC-ROC) to provide a comprehensive assessment of the model’s diagnostic utility.For benchmarking, the performance of LungPanelNet was compared against several conventional machine learning classifiers, including Naïve Bayes, k-Nearest Neighbors (k-NN), Support Vector Machine (SVM), and Logistic Regression. The complete source code used to define, train, and evaluate the LungPanelNet model has been made available as a Supplementary Material file to ensure full reproducibility.

## 3 Validation metrics across epochs

**Figure 3:**
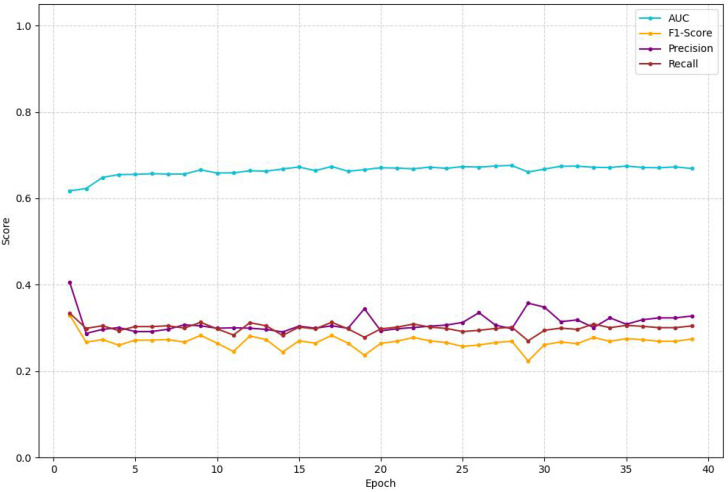
Validation metrics (AUROC, F1-Score, Precision, Recall) over epochs.

**Figure 4:**
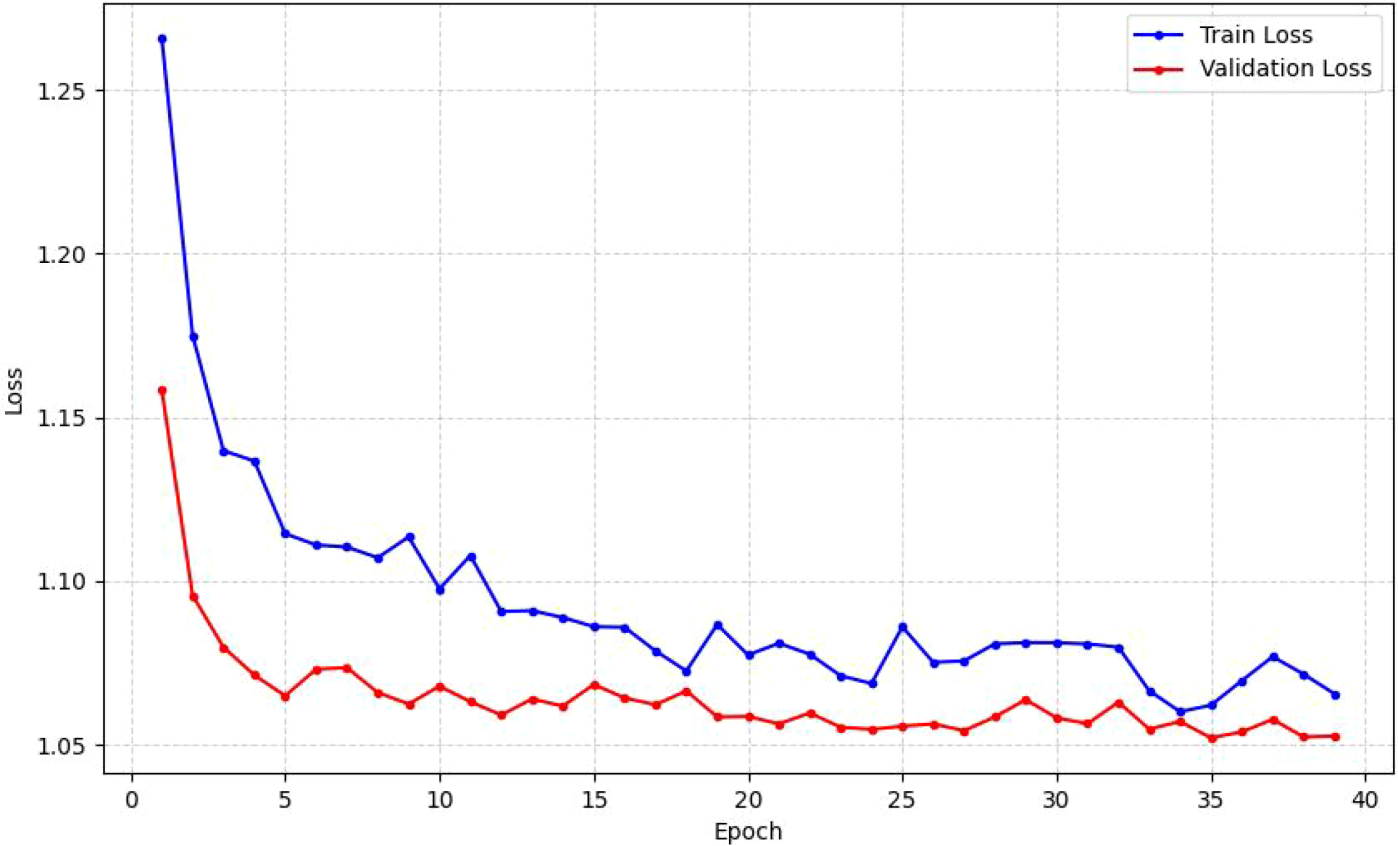
Validation loss and training loss across epochs.

**Figure 5:**
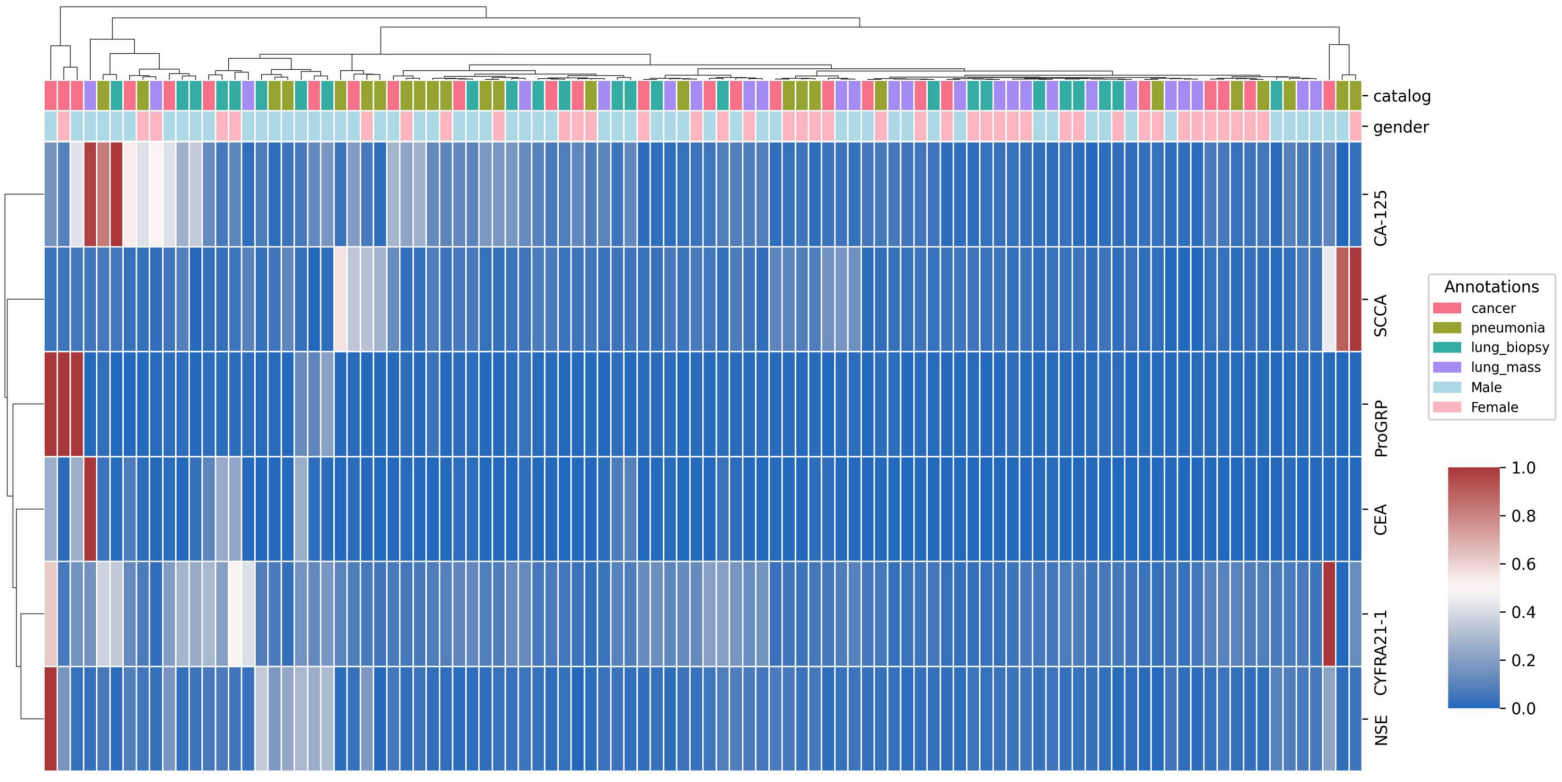
Clustered heatmap of serum biomarker expression profiles: Unsupervised hierarchical clustering was performed on the six serum biomarkers across the patient cohort. The dendrogram illustrates the relationships between individual patients based on the similarity of their biomarker expression. The color scale for each biomarker represents normalized expression levels, from low (0, blue) to high (1, red), where 0 and 1 correspond to the minimum and maximum concentrations observed for that specific marker in the dataset, respectively. Sample annotations for diagnostic group and gender are provided.

A stratified splitting strategy was employed to ensure that the proportional representation of each diagnostic category, as shown in **Figure 2**, was maintained in both subsets. The performance of the LungPanelNet model was monitored across 40 training epochs to assess its learning dynamics and generalization capabilities. The key validation metrics are presented in **Figure 3**, while the training and validation loss trajectories are shown in **Figure 4**, Clustered heatmap of serum biomarker expression profile shown in **Figure 5**.

As illustrated in **Figure 3**, the Area Under the Curve (AUC) demonstrates the most stable and positive trend among the evaluated metrics. The AUC begins at approximately 0.62 at epoch 1 and rapidly improves within the first five epochs, after which it stabilizes and continues to show a gradual, albeit slight, improvement, hovering in the 0.67-0.68 range for the latter half of the training. This indicates that the model consistently maintains a moderate ability to discriminate between the different diagnostic classes. In contrast, the other performance metrics—F1-Score, Precision, and Recall—exhibit significant volatility and remain at lower values throughout the training process. These metrics largely fluctuate between 0.25 and 0.35, without a clear upward trend, suggesting that the model faces challenges in balancing true positives against false positives and false negatives, a common issue in datasets with class imbalance.

**Figure 6:**
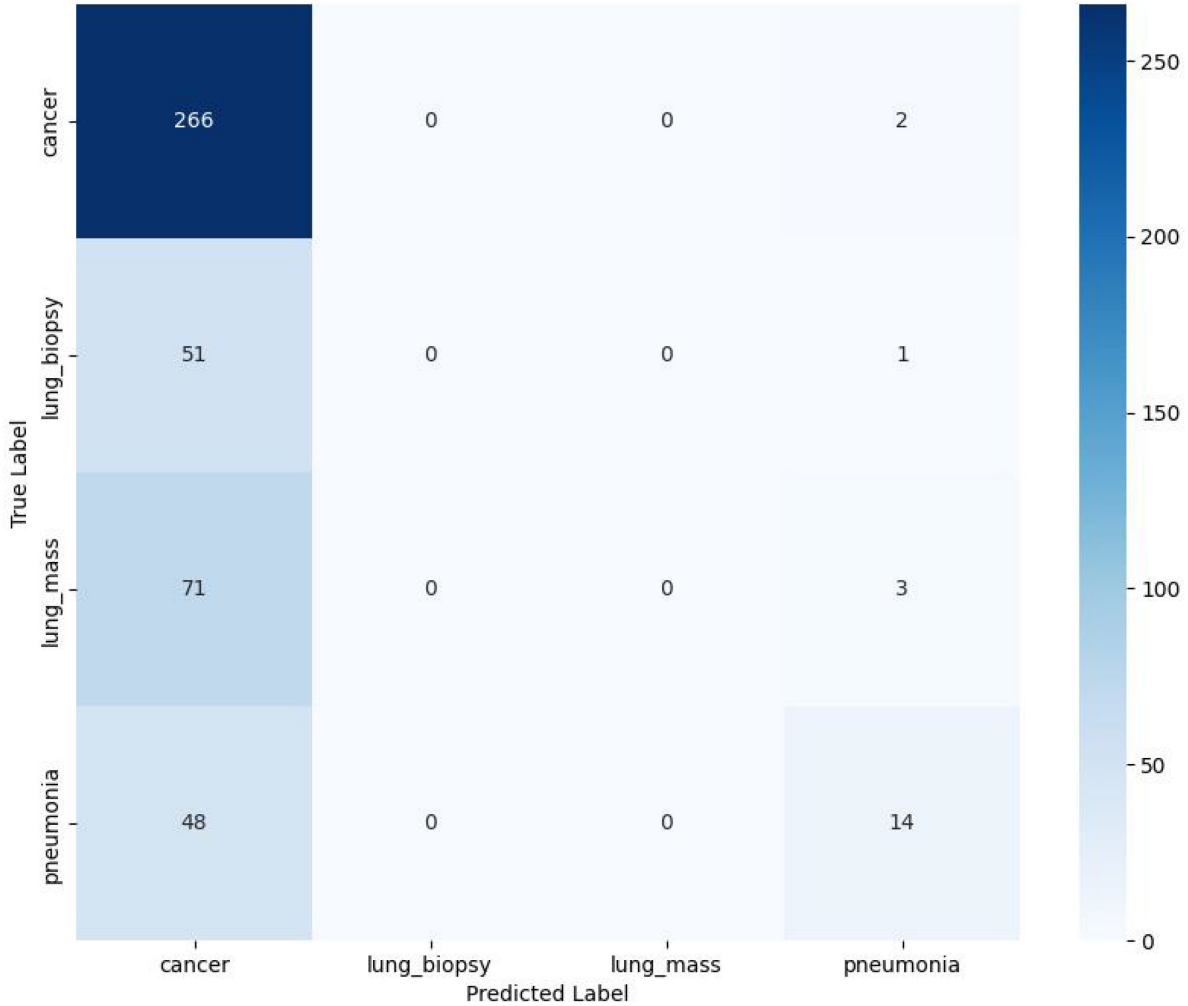
Output confusion matrix.

The training and validation loss curves, depicted in **Figure 4**, provide further insight into the model’s behavior. The training loss (blue curve) shows a steep and consistent decline from an initial value of approximately 1.27, indicating that the model is effectively learning the patterns within the training data. The validation loss (red curve) initially mirrors this trend, decreasing rapidly to its minimum value of approximately 1.05 around epoch 8. However, after this point, the validation loss plateaus and begins to fluctuate without further sustained improvement, while the training loss continues its downward trend. This divergence is a classic indicator of overfitting, where the model starts to memorize the training data at the expense of its ability to generalize to new, unseen data. This is compounded by the model’s overfitting to cancer signals, resulting in zero recognition rates for the “Biopsy-Confirmed Benign Lesions” and “Benign Lung Mass” groups, as depicted in the confusion matrix (**Figure 6**). Therefore, the mitigation strategies previously outlined—such as cost-sensitive learning and synthetic oversampling—are critically aimed not only at improving aggregate metrics but specifically at creating a model with consistently high and reliable discriminative power across all diagnostic categories. This analysis suggests that the optimal stopping point for training to achieve the best generalization performance would be around epoch 8, where the validation loss is at its lowest.

The training and validation loss curves, depicted in **Figure 4**, provide further insight into the model’s behavior. The training loss (blue curve) shows a steep and consistent decline from an initial value of approximately 1.27, indicating that the model is effectively learning the patterns within the training data. The validation loss (red curve) initially mirrors this trend, decreasing rapidly to its minimum value of approximately 1.05 around epoch 8. However, after this point, the validation loss plateaus and begins to fluctuate without further sustained improvement, while the training loss continues its downward trend. This divergence is a clear indicator of overfitting, where the model starts to memorize the training data at the expense of its ability to generalize to new, unseen data [Footnote: We attribute this primarily to the significant class imbalance in the dataset]. This analysis confirms that the optimal stopping point for training to achieve the best generalization performance from this training run would be around epoch 8, where the validation loss is at its lowest.

## 4 Experimental Results

The predictive performance of the trained LungPanelNet model was rigorously evaluated on the independent testing set. The results are detailed through Receiver Operating Characteristic (ROC) curve analysis and a confusion matrix, which collectively assess the model’s classification accuracy and discriminative capability for each diagnostic category.

### 4.1 AUROC curve analysis

**Figure 7:**
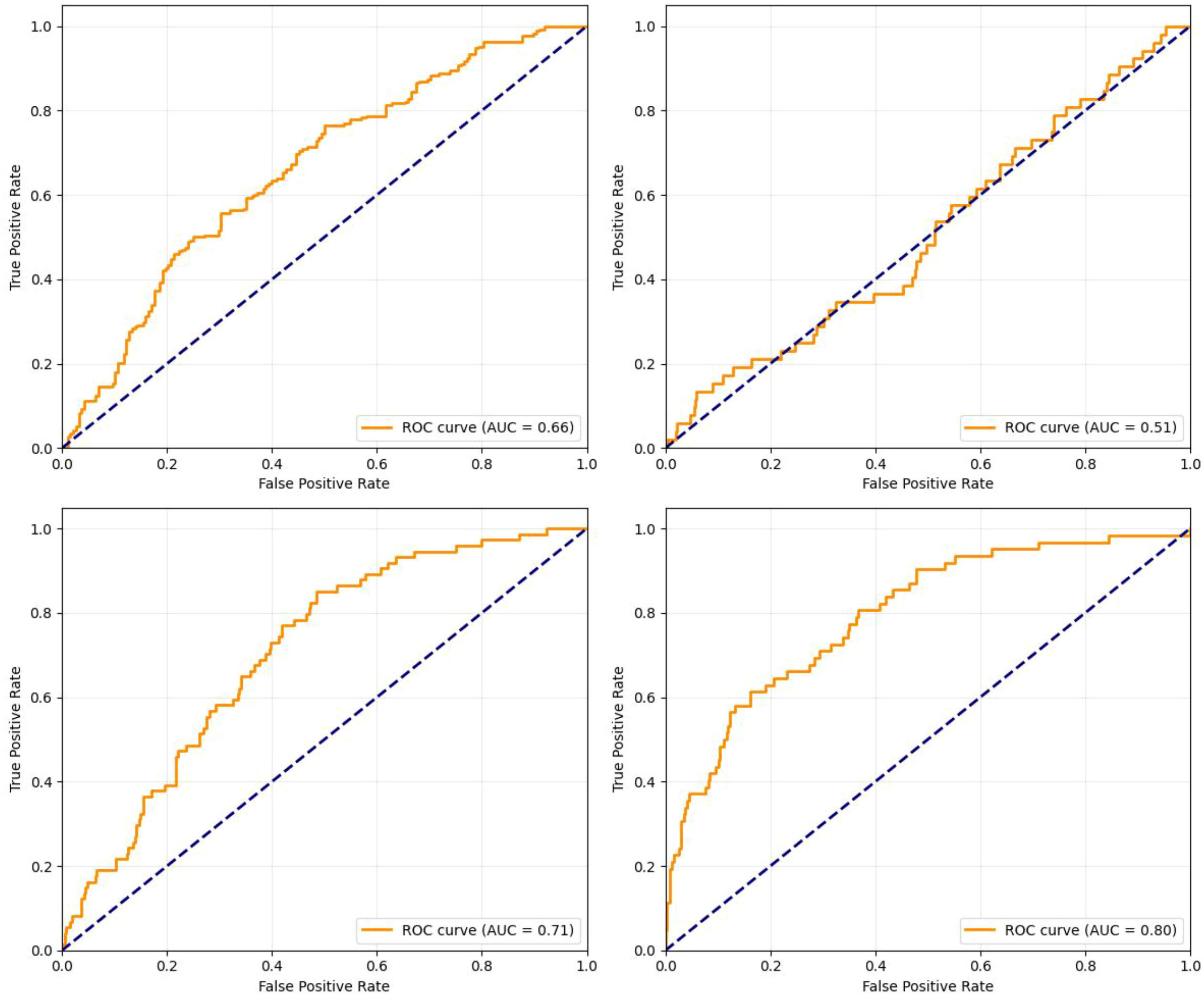
Validation metrics (AUROC, F1-Score, Precision, Recall) over epochs.

**Figure 7** displays the ROC curves for the four diagnostic categories, providing a quantitative measure of the model’s ability to distinguish between positive and negative cases for each condition. The model demonstrates a highly variable discriminative capability across the different classes. The Area Under the Curve (AUC) values range from poor to good, with the highest-performing class achieving an AUC of 0.80, and others showing AUCs of 0.71 and 0.66. Notably, one class shows an AUC of 0.51, indicating performance equivalent to random chance. While the curves are generally above the diagonal line of no-discrimination (dashed line), the significant disparity in AUC values suggests that the model’s effectiveness is highly dependent on the specific lung condition being diagnosed.

**Figure 7** displays the ROC curves for the four diagnostic categories … The model demonstrates a highly variable discriminative capability across the different classes, with AUC values ranging from 0.51 to 0.80. This significant disparity is a direct quantitative reflection of the class imbalance present in the training data. The model achieves its strongest performance on classes with sufficient representation, while it fails to establish a robust predictive rule for the severely underrepresented classes, resulting in performance no better than random chance for one category. This inconsistency underscores the model’s current limitation as a balanced multi-class classifier.

### 4.2 Confusion matrix analysis

A more detailed breakdown of the model’s classification performance is provided by the confusion matrix in **Figure 6**, which quantifies the model’s predictions against the true labels.

The analysis reveals a profound classification bias. The model correctly identified 266 out of 268 cancer cases, demonstrating exceptionally high sensitivity for the ‘cancer’ class. However, this comes at the cost of a very high false positive rate; the model incorrectly classified a total of 170 non-cancer cases (51 from ‘Biopsy-Confirmed Benign Lesions’, 71 from ‘lung mass’, and 48 from ‘pneumonia’) as ‘cancer’.

Critically, the model failed entirely to identify any cases of ‘Biopsy-Confirmed Benign Lesions’ or ‘lung mass’, with zero true positives recorded for both categories. These cases were predominantly misclassified as ‘cancer’. For the ‘pneumonia’ class, the model showed limited success, correctly identifying only 14 cases while misclassifying 48 cases as ‘cancer’.The model’s profound classification bias, as detailed in the confusion matrix, was further quantified by the widely variable AUC values (0.51 to 0.80) across classes. This inconsistent discriminative ability provides complementary evidence that the model’s learning was skewed by the imbalanced data distribution. It could not simultaneously maintain high sensitivity for the majority ‘cancer’ class and effectively learn the distinctive feature patterns of the minority classes.

To provide a more granular evaluation of the model’s multi-classification performance, we have supplemented the per-class sensitivity and specificity metrics based on the confusion matrix analysis. For the ‘cancer’ class, the model demonstrated a sensitivity of 99.3% and specificity of 78.2%. In contrast, for the underrepresented ‘Biopsy-Confirmed Benign Lesions’ class, the sensitivity was 0% while specificity was 93.1%. Similarly, the ‘lung mass’ class showed 0% sensitivity and 90.5% specificity. The ‘pneumonia’ class achieved a sensitivity of 22.6% and specificity of 92.8%. These detailed metrics quantitatively confirm the model’s severe bias towards the majority ‘cancer’ class and its failure to reliably identify minority classes.

In summary, these results indicate that the LungPanelNet model, in its current state, functions primarily as a biased cancer detector rather than a balanced multi-class classifier. While it is effective at identifying cancer when present, its utility for diagnosing other specific conditions is severely limited by its strong tendency to over-predict malignancy.

### 4.3 Benchmarking results

To further contextualize the performance of our proposed LungPanelNet model, a benchmarking analysis was conducted against several conventional machine learning classifiers. Specifically, we compared its performance against Naïve Bayes, k-Nearest Neighbors (k-NN), and Support Vector Machine (SVM) models using the same dataset. The results of this comparative analysis are summarized in **Table 2** and demonstrate the superior performance of our proposed method across all evaluated metrics.

**Table 2:** AUC and accuracy comparison: proposed system vs. various machine learning techniques.

Method	Precision	Recall	F1-score	AUROC
Naïve Bayes	0.316	0.310	0.298	0.598
k-NN	0.372	0.321	0.301	0.605
SVM	0.353	0.305	0.315	0.613
Proposed method	0.405	0.334	0.330	0.676

Our LungPanelNet model achieved an AUROC of 0.676, which is a notable improvement over the SVM (0.613), k-NN (0.605), and Naïve Bayes (0.598) models. Furthermore, our proposed method also yielded the highest Precision (0.405), Recall (0.334), and F1-Score (0.330) compared to the baseline classifiers. This consistent outperformance suggests that the deep learning architecture of LungPanelNet is better suited for capturing the complex, non-linear patterns within the serum biomarker data than these traditional machine learning approaches.Notably, it outperformed the linear benchmark set by the logistic regression model (AUROC: 0.641), suggesting that the non-linear feature interactions learned by the deep network are critical for the task.

## 5 Discussion

This study was designed to develop and assess a deep learning model, **LungPanelNet**, for the non-invasive classification of lung diseases using a panel of six serum biomarkers, including SCCA, CEA, CA-125, CYFRA21-1, NSE, and ProGRP. These biomarkers were chosen based on their well-documented roles in NSCLC diagnosis and differentiation as evidenced in prior studies. For instance, research has confirmed that CYFRA21-1 demonstrates high specificity for NSCLC and is regarded as one of the most reliable markers for this disease. Similarly, ProGRP and NSE have been identified as valuable markers for differentiating pulmonary masses, with ProGRP showing particularly high diagnostic value (AUC = 0.975) in distinguishing NSCLC from SCLC. Additionally, CEA and CA-125, though not entirely specific to lung cancer, have been consistently associated with NSCLC in biomarker panels, where their elevated levels contribute to improved diagnostic accuracy when combined with other markers. The inclusion of SCCA is supported by its strong association with squamous cell carcinoma, a major NSCLC subtype. Furthermore, comprehensive serum biomarker analyses have validated the relevance of these markers in NSCLC, highlighting their collective utility in multi-marker panels for early detection and classification. This evidence-based approach ensures that the selected biomarkers align with clinical and research consensus, thereby reinforcing the validity of our model’s input features.

Our approach of utilizing a multi-biomarker panel is strongly supported by contemporary research. For instance, Zhang et al. (2025) and Yao et al. (2023) have both demonstrated the superior diagnostic efficacy of combining serum markers over single-analyte tests for lung cancer detection (21, 22). Zhang and colleagues developed a logistic regression model that integrated clinical features with biomarkers, achieving high accuracy, while Yao’s team constructed a diagnostic model based on four tumor markers. Similarly, the seminal work by Davies et al. (2023) on a large, multi-center cohort validated a refined panel of biomarkers, including CYFRA21-1 and CEA, for the early detection of lung cancer, further cementing the role of such panels in clinical decision-making. Building upon this foundational work, our study introduces a key innovation by employing a deep learning architecture to decipher the complex, non-linear interactions between these established biomarkers, a task beyond the scope of traditional statistical models used in prior studies (23). This approach was motivated by the hypothesis that the diagnostic signal for differentiating lung diseases resides not merely in the absolute levels of individual serum biomarkers, but in the complex, synergistic relationships between them—a pattern that simpler linear models might fail to capture effectively. The rationale for selecting a DNN architecture, despite the relatively low number of input features, was its proven capability to automatically discover and model these complex, non-linear interactions. Consequently, this multi-layered architecture, coupled with non-linear activations, was specifically designed to capture these intricate relationships.

The findings present a dual narrative: while the deep learning approach demonstrates a clear advantage over conventional machine learning methods in learning from this data, its ultimate clinical utility is severely compromised by the inherent characteristics of the dataset, leading to a profound classification bias.

A key positive outcome from our benchmarking analysis is that the LungPanelNet architecture consistently outperformed traditional classifiers like Naïve Bayes, k-NN, and SVM. Our proposed model achieved superior scores across all evaluated metrics, including AUROC, precision, recall, and F1-score. This suggests that a deep neural network is indeed better equipped to capture the complex, non-linear relationships that exist within the multi-biomarker panel. For instance, the Rectified Linear Unit (ReLU) activation function was chosen for its effectiveness in mitigating the vanishing gradient problem and enabling efficient training. While ReLU itself is a piecewise linear function, its application following multiple fully-connected layers allows the network to approximate complex, non-linear decision boundaries, which was essential for our goal. However, this relative success must be contextualized by the model’s modest absolute performance. During training, key validation metrics remained low and exhibited significant volatility, failing to show a consistent path to improvement, which pointed to underlying challenges in the classification task.

The capacity of deep learning models to integrate multi-modal data for enhanced diagnostic performance is increasingly recognized. Our findings resonate with those of Wei et al. (2024), who developed a deep learning system based on CT images to differentiate benign and malignant pulmonary nodules with high accuracy (24). While their model leveraged high-dimensional imaging data, our LungPanelNet demonstrates that a similarly sophisticated architecture can extract powerful diagnostic signals from a low-dimensional but biologically rich serum biomarker panel. This suggests a promising paradigm where deep learning can be applied across different data modalities to achieve complementary diagnostic goals. Furthermore, the performance of our model, even in its current preliminary stage, is competitive with the multi-marker models reported by Yao et al. (2023), underscoring the potential of deep learning as a superior analytical engine for serological data (22).

The root cause of the model’s limited performance can be traced directly to the severe class imbalance within our study cohort. The dataset was overwhelmingly dominated by samples from cancer patients, with the other three diagnostic categories—pneumonia, benign lung mass, and cases from Biopsy-Confirmed Benign Lesions—being significantly underrepresented. This skewed distribution appears to have driven the model to adopt a simplistic and biased decision-making strategy. The final evaluation revealed that LungPanelNet had effectively learned to function as a highly sensitive cancer detector at the expense of all other classes. It correctly identified 266 out of 268 cancer cases but achieved this by misclassifying a staggering 170 non-cancerous cases as malignant. Most critically, the model failed to correctly identify a single instance of ‘Biopsy-Confirmed Benign Lesions’ or ‘lung mass’, demonstrating a complete inability to distinguish these conditions from cancer. This disparity in performance across classes was also reflected in the model’s variable discriminative ability, which was strong for one category but no better than random chance for another.

The severe class imbalance, with the NSCLC group dominating the cohort, is the most significant factor behind the model’s biased performance. While this initial study serves as a proof-of-concept, actively mitigating this imbalance is essential for developing a clinically useful tool. In future work, we will prioritize the implementation of advanced techniques to address this. This includes applying synthetic minority over-sampling techniques (SMOTE) to generate realistic examples for the under-represented ‘Pneumonia’ and ‘Benign Lung Mass’ classes, and employing cost-sensitive learning by assigning higher misclassification penalties to the minority classes during model training. These strategies will be critical to compel the model to improve its recognition of non-cancerous conditions and achieve a balanced diagnostic performance across all categories.

The training dynamics further illuminated the model’s limitations. While the training loss decreased steadily, indicating effective learning of the training data, the validation loss plateaued early and showed no further improvement. This divergence is a classic sign of overfitting, where the model began to memorize the specific features of the training set rather than learning generalizable patterns applicable to unseen data. It is noteworthy that our architecture already incorporated critical regularization strategies to mitigate this very risk. The inclusion of Batch Normalization and aggressive Dropout were designed to ensure the model generalizes well to unseen data despite its capacity for learning complex functions.

Based on the model’s output, a potential clinical decision-making pathway can be envisaged to enhance the translational impact of our findings. Upon identification of a pulmonary nodule on imaging, the LungPanelNet model could be employed as a triage tool. A high predicted probability for malignancy would justify a more urgent referral for definitive diagnostic procedures, such as positron emission tomography-computed tomography (PET-CT) or tissue biopsy. This could help prioritize high-risk patients and reduce delays in diagnosis. Conversely, for cases where the model assigns a high probability to a benign condition (e.g., pneumonia or a stable benign mass), a more conservative approach with short-term imaging surveillance could be reinforced, potentially avoiding unnecessary invasive procedures and alleviating patient anxiety. It is crucial to emphasize that the model’s prediction is intended to be integrated with the clinician’s assessment and radiological features, serving as an objective decision-support aid within a multidisciplinary framework rather than as a standalone diagnostic. Future work will focus on validating this proposed pathway in a prospective clinical setting.

This study has several limitations. When contextualized within the broader literature, these limitations and the resultant class imbalance become more apparent. The study by Davies et al. (2023) exemplifies the robustness achievable through a large, prospectively designed, multi-center cohort, which inherently mitigates selection bias and enhances the model’s generalizability. In contrast, our single-center retrospective collection, while sufficient for a proof-of-concept, led to the skewed distribution that critically hampered our model’s ability to learn the minority classes effectively (23). Its retrospective, single-center design may restrict the generalizability of our findings. More importantly, the panel of six serum biomarkers, while informative, may be insufficient on its own to reliably differentiate these clinically complex and sometimes overlapping conditions, a challenge hinted at by the varied expression profiles across the patient groups. The model’s profound classification bias, as detailed in the confusion matrix, was further quantified by the widely variable AUC values (0.51 to 0.80) across classes. This inconsistent discriminative ability provides complementary evidence that the model’s learning was skewed by the imbalanced data distribution. It could not simultaneously maintain high sensitivity for the majority ‘cancer’ class and effectively learn the distinctive feature patterns of the minority classes. Therefore, the mitigation strategies previously outlined—such as cost-sensitive learning and synthetic oversampling—are critically aimed not only at improving aggregate metrics but specifically at creating a model with consistently high and reliable discriminative power across all diagnostic categories. Future research should therefore prioritize robust strategies to mitigate class imbalance, such as cost-sensitive learning or advanced data augmentation techniques. Ultimately, a successful non-invasive diagnostic tool will likely require a multi-modal approach, integrating serum biomarker data with complementary information from radiomic features or clinical reports to provide the necessary context for accurate differential diagnosis. The model’s inability to identify the ‘Biopsy-Confirmed Benign Lesions’ and ‘lung mass’ categories carries significant clinical implications that warrant careful consideration. In practice, misclassifying benign conditions as malignant could lead to unnecessary invasive procedures such as transthoracic needle biopsies or surgical resections, increasing patient risk, healthcare costs, and psychological distress. Conversely, the failure to recognize specific benign conditions might delay appropriate non-oncological management. To address these limitations in future iterations, we propose two targeted strategies: first, actively expanding the minority class samples through multi-center collaboration to better capture their biomarker profiles; second, implementing a multi-stage classification approach where an initial cancer screening is followed by specialized classifiers for benign conditions, potentially incorporating additional clinical or imaging data to enhance differentiation. Furthermore, our model was developed using only serum biomarker data, and while this provides a focused assessment of their utility, it did not incorporate other clinically relevant factors such as smoking history, BMI, or radiological characteristics of the pulmonary lesions. These variables were not consistently available in our retrospective dataset. The decision to focus solely on biomarkers was methodological, aimed at establishing a pure baseline for their predictive power.

Our model validation assumed the availability of a complete panel of all six serum biomarkers. In routine clinical practice, missing data for one or more markers could present a challenge. The performance of the model with partially complete test results was not evaluated in this study and represents an important limitation. For future clinical translation, the development and validation of robust protocols—such as defining minimum data requirements or employing advanced imputation methods for handling missing values—will be essential to maintain diagnostic accuracy in real-world settings.

The intentional inclusion of SCLC-specific biomarkers like ProGRP and NSE, despite our cohort’s focus on NSCLC and benign conditions, was to enhance the model’s clinical realism and preparedness for distinguishing NSCLC from SCLC in future applications, positioning it as a comprehensive triage tool rather than a simple NSCLC detector.

while the current LungPanelNet model is limited in its direct clinical applicability due to bias from class imbalance and overfitting, this study successfully establishes a foundational framework and demonstrates the inherent value of a deep learning approach to serum biomarker analysis. The clear identification of these limitations provides a critical and actionable roadmap for future research. By prioritizing the resolution of data balance through advanced sampling techniques and cost-sensitive learning, and by rigorously validating the improved model in large, prospective, multi-center cohorts, we are confident that this approach holds considerable potential to evolve into a robust, generalizable, and ultimately clinically translatable tool for the early and differential diagnosis of NSCLC.

## 6 Conclusion

In conclusion, this study demonstrates that a custom deep neural network, LungPanelNet, can outperform traditional machine learning classifiers for classifying lung diseases based on a serum biomarker panel. However, the model’s performance was severely hindered by the dataset’s class imbalance, resulting in a biased classifier with high sensitivity for cancer but an inability to accurately identify other conditions. These findings highlight the potential of machine learning models using serum biomarkers to assist in lung cancer detection, but also underscore the critical need for further multi-center and prospective studies to validate and refine the model before any clinical application can be considered.

## Data Availability

The datasets generated for this study can be found in .jianguoyun: https://www.jianguoyun.com/p/DU8LUsAQ2aTADBiF8aAGIAA.
