# Supplementary figures and images for: LungPanelNet: a machine learning-based approach for the early prediction and differentiation of non-small cell lung cancer
Source: Front Oncol. 2026 Jan 13;15:1702589. doi: 10.3389/fonc.2025.1702589 (PMC12834828; doi:10.3389/fonc.2025.1702589)

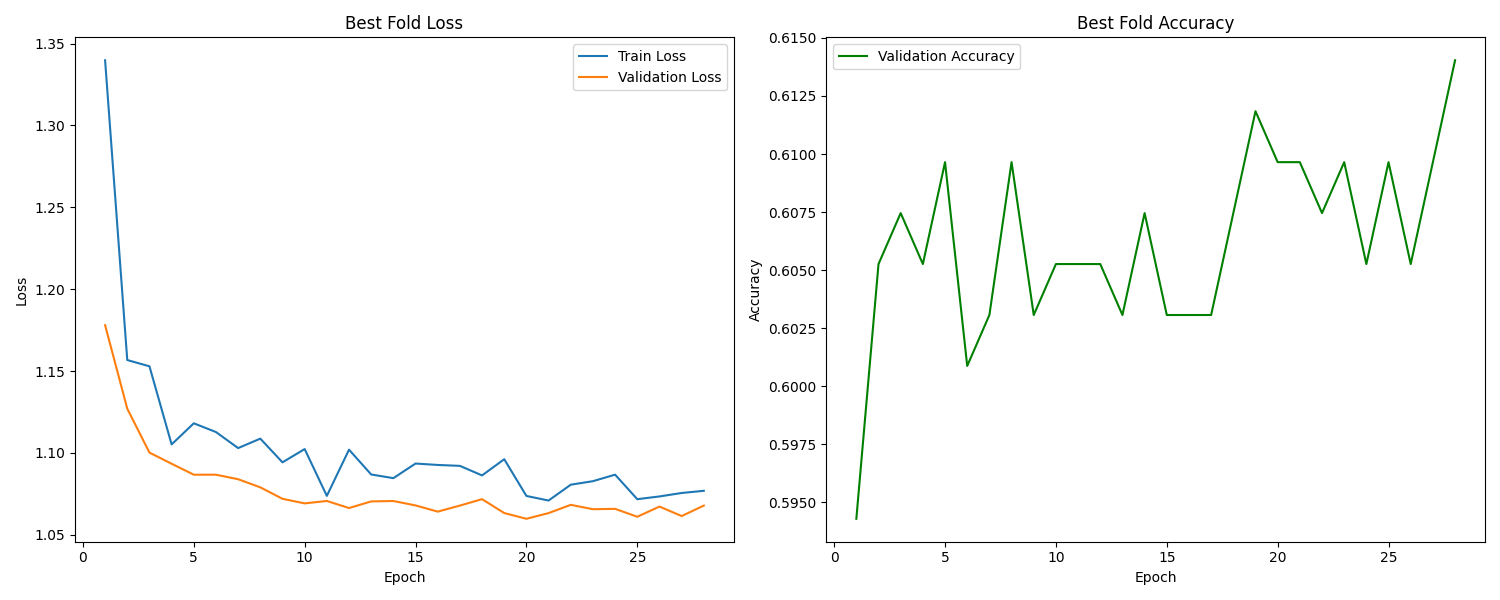

Supplement: Supplementary file 1 [file Image1.png]

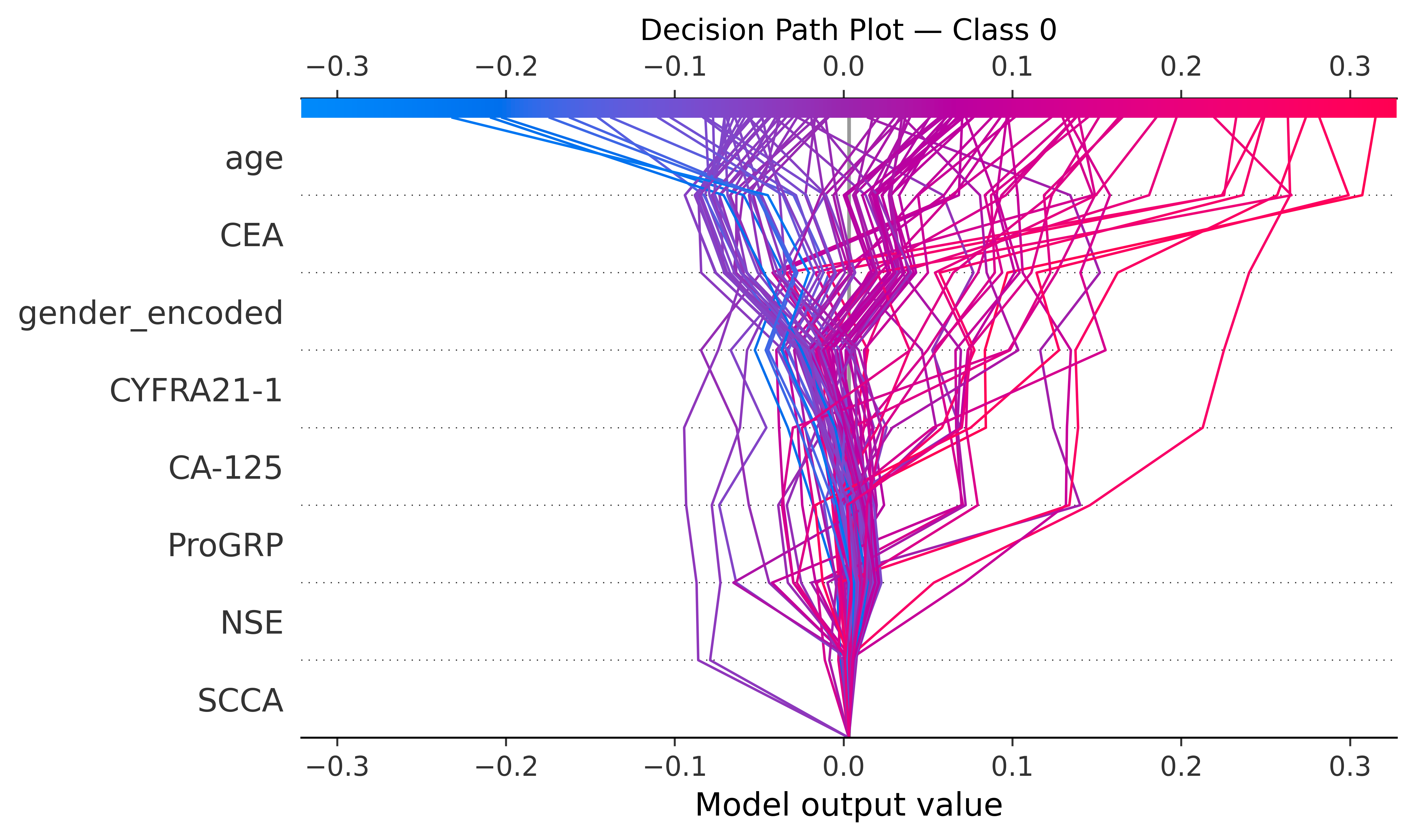

Supplement: Supplementary file 2 [file Image2.png]

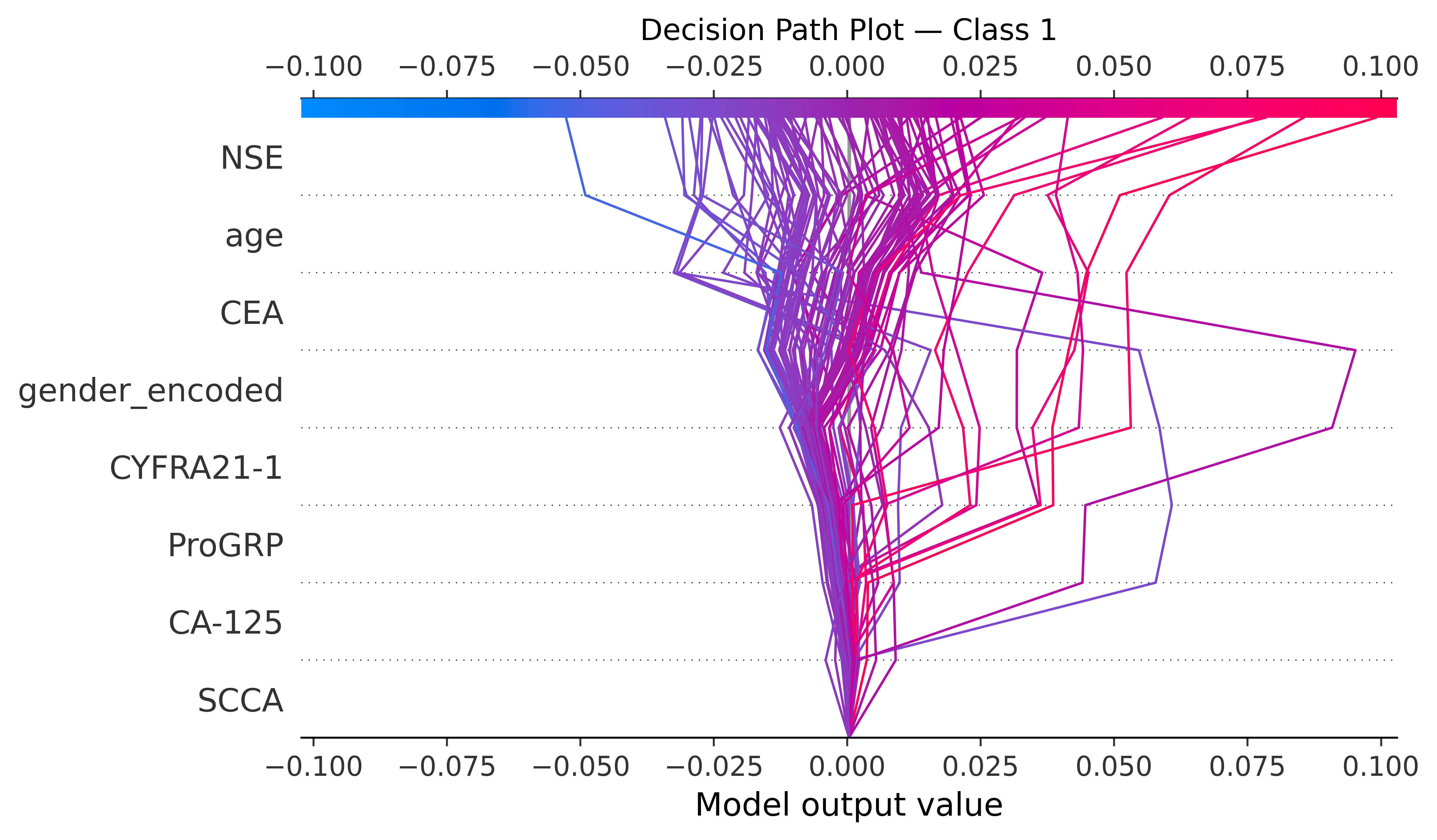

Supplement: Supplementary file 3 [file Image3.png]

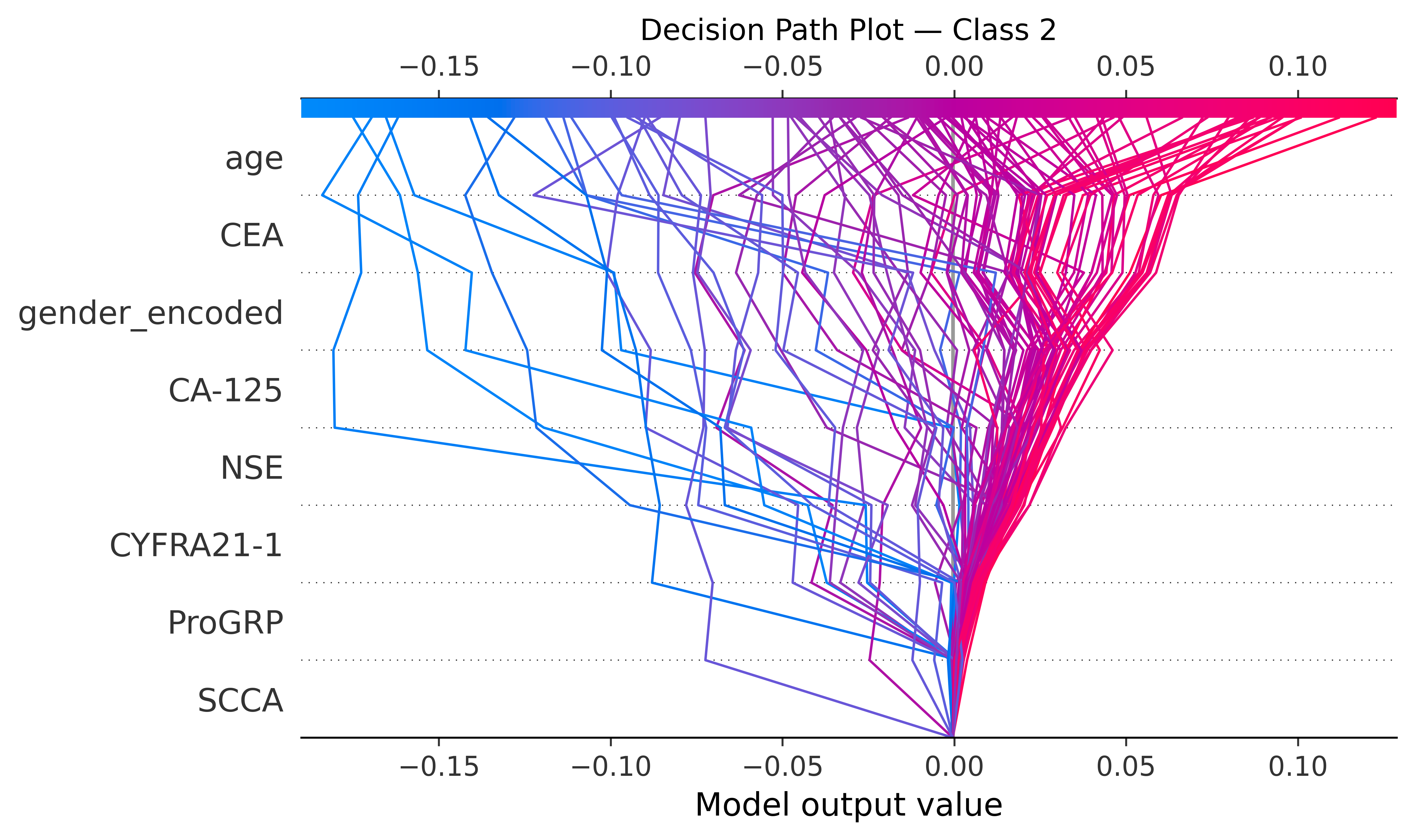

Supplement: Supplementary file 4 [file Image4.png]

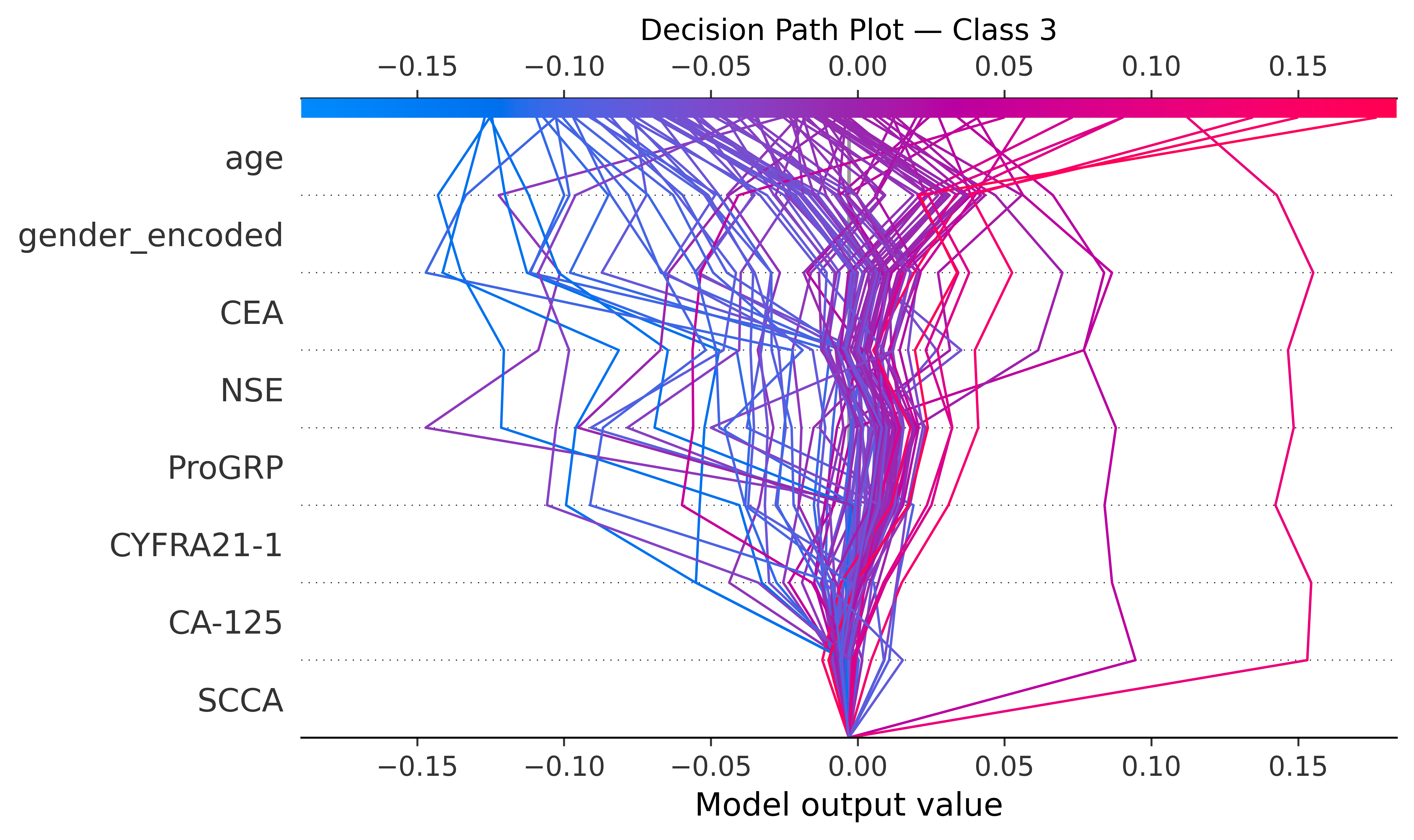

Supplement: Supplementary file 5 [file Image5.png]

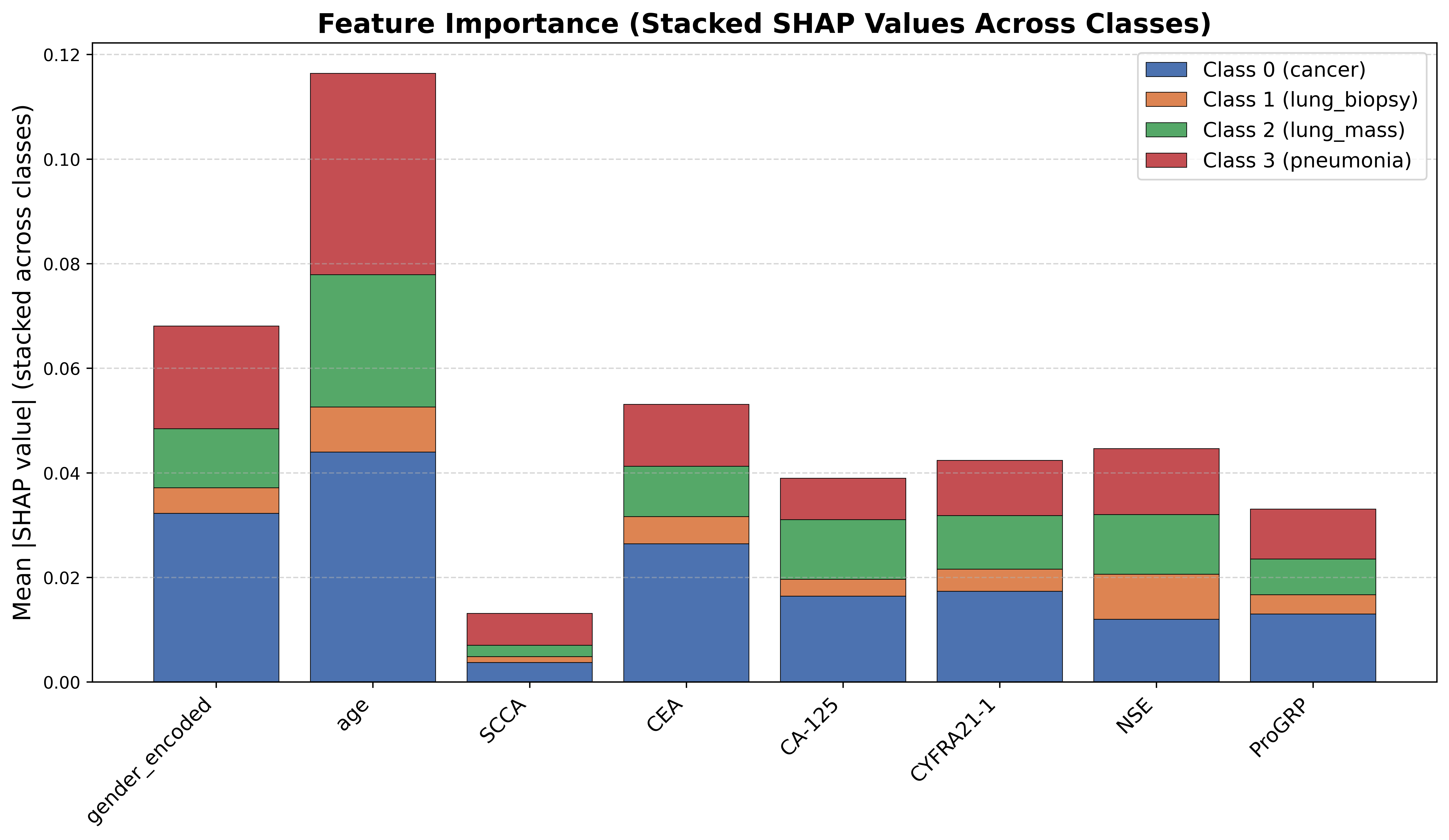

Supplement: Supplementary file 13 [file Image13.png]
